# Screening for prostate cancer: Controversy? What controversy?

**DOI:** 10.3747/co.v16i3.459

**Published:** 2009-05

**Authors:** R.J. Ablin, M.R. Haythorn

The purpose of screening is to identify preclinical and asymptomatic cases of a disease in a population at risk—as opposed to making a diagnosis based on a patient’s presentation with symptoms and disease.

The use of the prostate-specific antigen (psa) test and a digital rectal examination (dre) to screen for prostate cancer—and whether such use can reduce prostate cancer–specific mortality—has universally been regarded as controversial despite the publication of more than 1000 articles in the medical and scientific literature. In fact, in spite of national guidelines by the Canadian Task Force on Preventive Health Care, which do not recommend psa screening for prostate cancer in asymptomatic men^1^, 72% of Canadian men in a national survey reported receiving psa screening^2^. And, quoting from a publication by investigators from the Centre for Chronic Disease Prevention and Control, Public Health Agency of Canada, “we wait for randomized controlled trial evidence of the effectiveness of psa screening in reducing mortality due to prostate cancer”^2^

The long anticipated wait for Canadians and others ended, in part, in March of this year with the publication of interim data from 7–10 years of follow-up in two large ongoing randomized screening trials^3^,^4^. The trials were thought to be decisively designed to resolve the controversy over whether screening for prostate cancer reduces mortality. The Prostate, Lung, Colorectal and Ovarian (plco) trial, started in 1993 in the United States, and the European Randomized Study of Screening for Prostate Cancer (erspc), started in 1994, respectively enrolled nearly 77,000 and 182,000 men randomly assigned to receive screening or not.

The plco trial^3^ found no difference in mortality from combined screening with the psa test and dre through 10 years; the erspc trial 4 reported that psa screening without dre was associated through 9 years with a 20% reduction in mortality from prostate cancer. With due consideration to the stated 20% reduction in mortality, it is noted that 1410 men needed to be screened and 48 men treated to prevent 1 cancer death, thus overtreating 47 men.

In the interim since the publication of the two trials, several possible explanations have been offered for the absence of a reduction in mortality in the plco trial and in comparison with the difference seen in the erspc trial. The truth of the matter is the psa test should not be used at all in screening for prostate cancer in the manner it has heretofore been used. Prostate-specific antigen, discovered in 1970^5^,^6^, is a normal component of the prostate. It is not cancer-specific. Rather, it is present in the normal, benign, and malignant prostate 7. When the U.S. Food and Drug Administration (fda) approved the psa test in 1994 for screening, the approval was not based on rigorous study of the specificity or sensitivity of the test. The fda never looked at the benefits and risks beyond a 3.8% detection rate compared with a 0.8%–1.4% detection rate for dre.

The ability of the psa test to identify men with prostate cancer is slightly better than that of flipping a coin. Further, prostate cancer is an age-related disease, and the psa test may merely precipitate a biopsy, wherein, related to age, the biopsied individual may or may not have cancer—a finding that, according to some, may be related to “how hard it (i.e., cancer) is looked for” 8. With approximately 45%–80% of men between the ages of 50 and 75 years of age (the age-range of the men in the plco and erspc trials) possessing indolent or clinically insignificant cancers 9, the detection of prostate cancer by psa test, given its absence of cancer specificity, has more than likely been a serendipitous observation. Therefore, based on the initial studies of psa 5–7 and the subsequent observations of one of us (RJA), the interim results from the plco and erspc trials showed exactly what we would have expected.

Although the erspc study was “designed to show a 25% statistically significant reduction in possible prostate cancer mortality after screening” 10, it found only a 20% reduction in mortality. In addition, if the inconsistencies between erspc study sites—methodology, frequency of screening, and psa cutoff points (that is, 3.0 vs. 4.0) as an indication for biopsy—are looked at, the data may reasonably be questioned. With further reference to the 20% reduction in prostate cancer mortality between the screened and the unscreened groups, the assumption is made that, even with strict adherence to the criteria for the determination of cause of death in randomized screening trials for prostate cancer 10, given the small number of deaths in the screened (*n* = 261) and unscreened (*n* = 363) groups, with 20% being a relatively very small number, the determination could have included those very few cases in which the reviewers—that is, the cause-of-death committee—came to a different decision on cause of death.

Parenthetically, it is of interest to note that, in the absence of the inclusion of a dre as part of their screening protocol, the erspc trial did not follow the fda-approval guidelines, which state that the psa test “was approved for use in conjunction with a digital rectal exam”^11^.

In the face of the facts and the interim results of the plco and erspc trials, organizations that remain in support of screening for prostate cancer, particularly the American Urological Association, whose president, John Barry, has stated that “psa testing for prostate cancer remains a valuable screening tool and should be appropriately offered to men” 12, need to re-examine the dictum *Primum non nocere*: “First do no harm.”
